# Passive stabilization of low-power laser beams in soft hydrogels with parabolic-index profile for optogenetic neural stimulation: a phase analysis

**DOI:** 10.1039/d6ra03790a

**Published:** 2026-07-03

**Authors:** Hadi Rahimi

**Affiliations:** a Department of Physics, Shab.C., Islamic Azad University Shabestar Iran h_rahimi@iau.ac.ir

## Abstract

Optogenetic stimulation of deep brain regions is limited by rapid light divergence in soft tissue, mechanical damage from rigid optical fibers, and the impracticality of active beam control systems that require external power. This study introduces a passive, biocompatible solution based on soft parabolic-index hydrogels that stabilize the phase of propagating Gaussian beams without any external energy. Using beam propagation method simulations and phase-resolved analysis, we demonstrate three main findings: first, the proposed hydrogel maintains periodic focusing and defocusing cycles along the propagation path. Second, the transverse wavenumber becomes discrete and quantized, which confirms preservation of spatial coherence. Third, the optical phase undergoes periodic resetting to its initial value after each oscillation cycle, ensuring predictable beam focus at regular intervals. Furthermore, we show that the phase-intensity correlation stabilizes rapidly after initial propagation, and that the phase accumulation rate can be tuned by adjusting the gradient coefficient of the hydrogel. These findings establish that graded-index hydrogels can function as passive, implantable optical waveguides for delivering phase-stable, focused light to precise neural targets. This technology directly resolves the three major obstacles in optogenetic neural stimulation devices: light delivery, tissue damage, and reliance on external power.

## Abstract 2

Periodic focusing and phase resetting of a Gaussian beam in a GRIN hydrogel. The beam width oscillates periodically, and the phase returns to its initial value at each focal plane.

## Graphical abstract

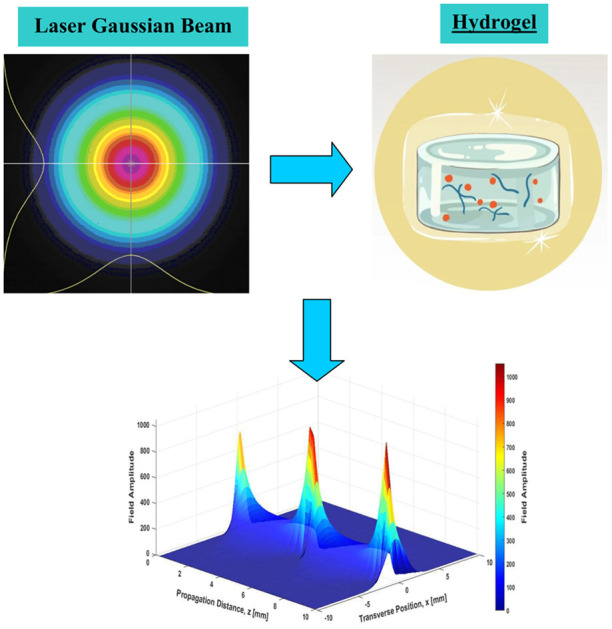

## 1 Introduction

Optogenetics is a revolutionary technique in neuroscience and biomedical research that enables precise control of neural activity using light.^1,2^ By genetically modifying specific populations of neurons to express light-sensitive proteins called opsins, researchers can either activate or inhibit these cells with millisecond precision simply by shining light of appropriate wavelengths.^3^ This powerful technology has opened new avenues for understanding neural circuits and treating neurological disorders such as Parkinson's disease,^4^ epilepsy,^5^ chronic pain,^6^ and even for restoring vision or hearing.^7,8^ The fundamental mechanism relies on microbial opsins which are light-gated ion channels that respond to specific wavelengths. When light of the correct wavelength strikes these opsins, they undergo a conformational change that opens ion channels or activates ion pumps, which leads to either depolarization (excitation) or hyperpolarization (inhibition) of the target neurons.^9^

Several recent studies have advanced optogenetic technologies toward safer, more precise hardware flexibility, stimulation resolution, and long-term biocompatibility. Lee *et al.*^10^ achieved targeted optogenetic stimulation using thin, flexible polymer probes integrated with advanced micro-OLEDs, significantly reducing tissue damage. A large-scale, high-density microLED array for neural stimulation and recording was developed by Gu and colleagues,^11^ enabling broader circuit interrogation. Cho *et al.*^12^ introduced a fully bioresorbable hybrid opto-electronic implant that allows simultaneous electrophysiological recording and optogenetic stimulation, eliminating the need for surgical removal. In the context of nerve repair, wireless stimulation using MXene-chitosan photo-responsive conduits has been shown to enhance regeneration after optic nerve injury.^13^ Heavy metal-free photon upconversion nanoparticles were employed by Uji *et al.*^14^ to achieve *in vivo* optogenetics without toxicity concerns. Cellular-resolution optogenetics was used to reveal attenuation-by-suppression in visual cortical neurons,^15^ which demonstrates how fine spatial control can uncover new circuit mechanisms. Kienbacher and colleagues^16^ extended the optogenetic toolbox to oncology, targeting hallmark traits of cancer cells. Efficient and sustained optogenetic control of sensory and cardiac systems was demonstrated in a recent study,^17^ highlighting translational potential beyond neuroscience. A computational approach to optimizing two-photon holographic stimulation sites was introduced by Triplett *et al.*,^18^ improving precision and reducing off-target effects. Finally, the technical development of two-photon optogenetic stimulation and its potential application to brain–machine interfaces has been thoroughly reviewed.^19^

To address the key challenges of delivering light to the brain for optogenetic therapy such as maintaining sufficient intensity and minimizing tissue damage soft graded index (GRIN) hydrogels offer a promising passive and biocompatible solution by preventing beam divergence and eliminating the need for external power.^20,21^ This approach builds on recent advances in hydrogel-based optical waveguides, which have emerged as excellent alternatives to traditional glass fibers due to their tissue-like mechanical properties and optical transparency.^22^ Innovations such as temperature-adaptive hydrogel fibers for deep-tumor treatment,^23^ wet-spinning methods for low-attenuation core–sheath designs enabling deep-tissue cancer therapy and brain optogenetic stimulation,^24^ and microfluidic-fabricated conductive hydrogels combining light transmission with sensing capabilities^25,26^ have significantly expanded the potential of these materials. A broader review on hydrogel fibers further highlights their design and bionic applications in mimicking human tissues.^27^ Together, these developments position soft hydrogels as a practical and effective platform for chronic, implantable neural stimulation.

The present study aims to investigate, from a phase-dynamics perspective, how soft hydrogels with a parabolic-index profile can passively stabilize the phase of propagating laser Gaussian beams for optogenetic neural stimulation. To achieve this goal, we employ the beam propagation method, a numerical technique for simulating optical wave propagation in inhomogeneous media. By using MATLAB software, we systematically vary the gradient coefficient and the on-axis refractive index to determine their individual contributions to the phase resetting behavior. This parametric flexibility directly informs the design of future implantable GRIN hydrogel probes, as different neural targets may require different focusing periods and confinement strengths depending on their anatomical depth and the desired stimulation precision.

## 2 Methods and materials

### 2.1 Gaussian beams in parabolic media

Consider the propagation of an input Gaussian beam in a quadratic index medium with a refractive index profile given by:^28,29^1
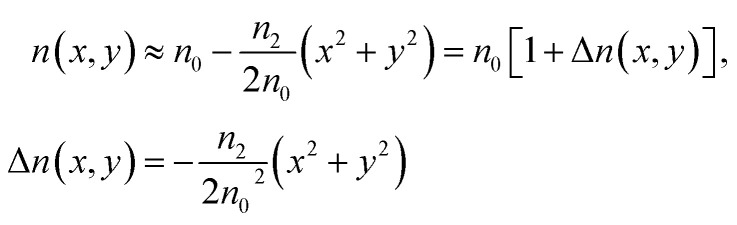
or equivalently, a permittivity profile:2
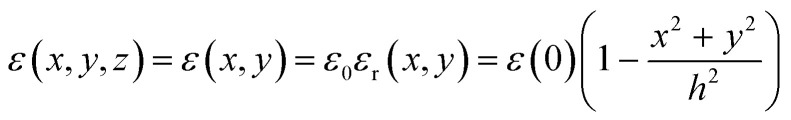
where *h* = *n*_0_/*n*_2_^1/2^, *n*_0_ is the on-axis refractive index, which is the maximum value of the refractive index at the center (*x* = 0, *y* = 0) of the quadratic medium, and *n*_2_ is the quadratic gradient coefficient, a positive constant that determines how rapidly the refractive index decreases away from the optical axis according to the parabolic profile. The input field at *z* = 0 is assumed to have a Gaussian amplitude profile as3
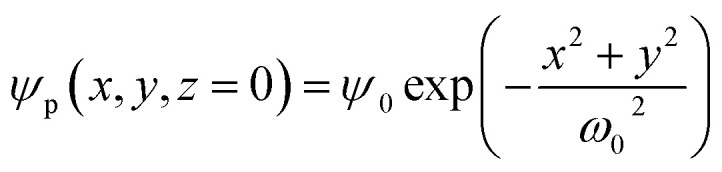
where *ω*_0_ is the initial beam waist or spot size at the input plane (*z* = 0). It represents the radius at which the Gaussian beam's electric field amplitude falls to 1/*e* of its maximum value at the beam center. Also, *ψ*_0_ is the peak amplitude of the complex electric field envelope at *z* = 0. Using the modal expansion approach and the approximate propagation constant for large *k*_0_*h*, we can have:4
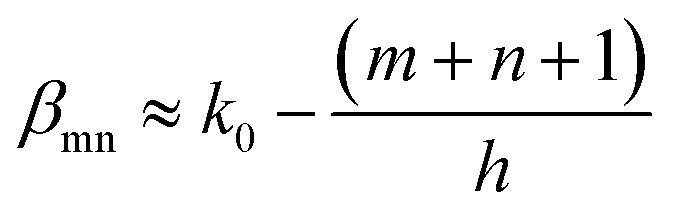


*β*_mn_ is the longitudinal propagation constant for a Hermite–Gaussian mode specified by non-negative integer indices *m* and *n*. The term *k*_0_ = 2π*n*_0_/*λ*_0_ is the wavenumber in a uniform medium with the on-axis refractive index *n*_0_. The expression (*m* + *n* + 1) represents the total order of the mode. This approximation, valid for *k*_0_*h* ≫ 1, shows that the propagation constant decreases linearly with the mode order, implying that higher-order modes travel with a lower phase velocity, which is a manifestation of intermodal dispersion in this weakly guiding parabolic waveguide.

The field at any propagation distance *z* can be obtained analytically *via* the superposition integral:^28,29^5
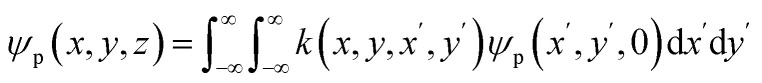
where *k*(*x*, *y*, *x*′, *y*′) is the parabolic medium's Green's function. Variables *x*′ and *y*′ represent the transverse coordinates at *z* = 0. In contrast, *x* and *y* are the coordinates in the output plane at a propagation distance *z* > 0. Physically, the *k*(*x*, *y*, *x*′, *y*′) describes how a unit-amplitude point source located at (*x*′, *y*′) in the input plane contributes to the complex field amplitude at the observation point (*x*, *y*) after propagating a distance *z*. Substituting the input Gaussian and the Green's function yields the closed-form expression:6
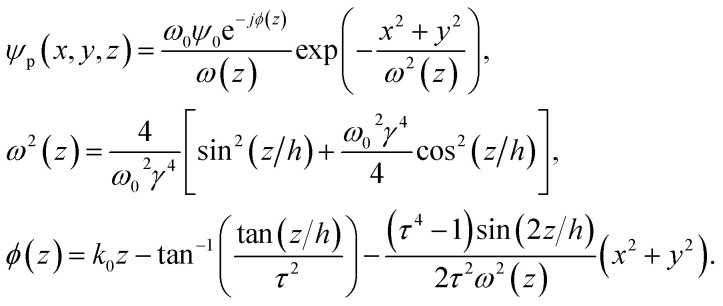
where 
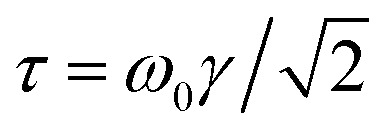
, *γ* = (*k*_0_/*h*)^1/2^ and *k*_0_ = *ω*_0_[*µ*_0_*ε*(0)]^1/2^.

Gaussian beam remains Gaussian while propagating in a quadratic index medium, but its width oscillates periodically with a period *z*_m_ = π*h*. The beam alternately focuses and defocuses along the propagation direction. If the input waist *ω*_0_ matches the fundamental mode waist of the medium:7
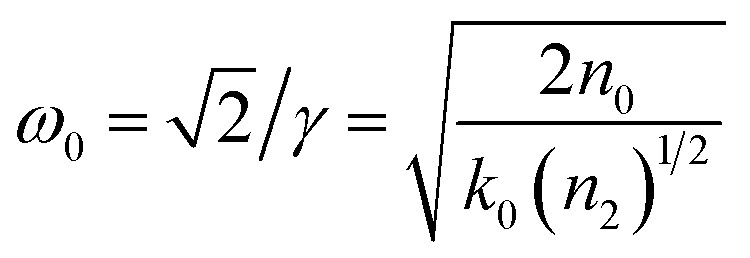


Then only the fundamental Hermite–Gaussian mode is excited, and the beam propagates without change in width. Otherwise, the beam exhibits periodic oscillations in its spot size which demonstrates the waveguide-like property of the quadratic index medium.^28,29^

### 2.2 Hydrogel compositions

Hydrogels are 3D polymer networks that hold large amounts of water, making them useful for biomedical and optical applications due to their biocompatibility, tunable mechanics, and transparency. They are made from natural or synthetic polymers. Natural hydrogels (*e.g.*, hyaluronic acid, gelatin, alginate) are non-toxic, biocompatible, biodegradable, and can support tissue healing, but they lack strength and stability. Synthetic hydrogels (*e.g.*, PVA, PEG, sodium polyacrylate, PVP) are generally stronger and more stable, and many are also suitable for medical use.^30,31^ Hydrogels are mostly water, so their refractive index depends on how much water and polymer they contain. Using the simple rule of mixtures: *n*_0_ = *n*_water_ × *φ*_water_ + *n*_polymer_ × *φ*_polymer_, where *φ*_water_ + *φ*_polymer_ = 1. The *φ* is the volume fraction of polymer and water. For a typical hydrogel with 60% water (*n*_water_ ≈ 1.33) and 40% polymer (*n*_polymer_ ≈ 1.6), we obtain *n*_0_ ≈ 1.45.

On the other hand, when the hydrogel swells, its size and refractive index change, but the focusing period of the beam stays almost the same because the key parameters change together. If there is a small error during the fabrication of the hydrogel, the focus point of the light beam shifts only a little, which is not a problem for most practical applications. Also, hydrogels are transparent because the pores inside them are much smaller than the wavelength of light.^32^ So light passes through them easily, and even after several centimeters, most of the light still remains, more than enough for applications like optogenetic stimulation.

### 2.3 Preparation of GRIN hydrogels

Graded-index profiles in hydrogels can be fabricated using established techniques such as diffusion-controlled photopolymerization,^33^ femtosecond laser-induced refractive index modification,^34^ and two-photon polymerization.^35^ A specific example is the PEGDA–gelatin hydrogel system, which demonstrates suitable optical and mechanical properties for GRIN applications.^36^ To prevent the gradient from degrading over time, additional crosslinking can be introduced after gel formation.^37,38^

## 3 Results and discussion

Here, we simulate numerically the propagation of low-power Gaussian beams in a biocompatible hydrogel medium characterized by a parabolic (quadratic) refractive index profile. “Low-power” in this work refers to laser powers that do not cause heating or damage in biological tissue. Based on standard safety guidelines, this range is typically between 0.1–1000 mW.^39^ On-axis refractive index is *n*_0_ = 1.5 and quadratic gradient coefficient is *n*_2_ = 0.01 mm^−2^. The wavelength of input Gaussian beam is *λ*_0_ = 0.633 µm (corresponding to a common He–Ne laser) with waist *ω*_0_ = 0.5 mm. The computational domain spanned *L* = 10 mm in the transverse direction.

**Fig. 1:**
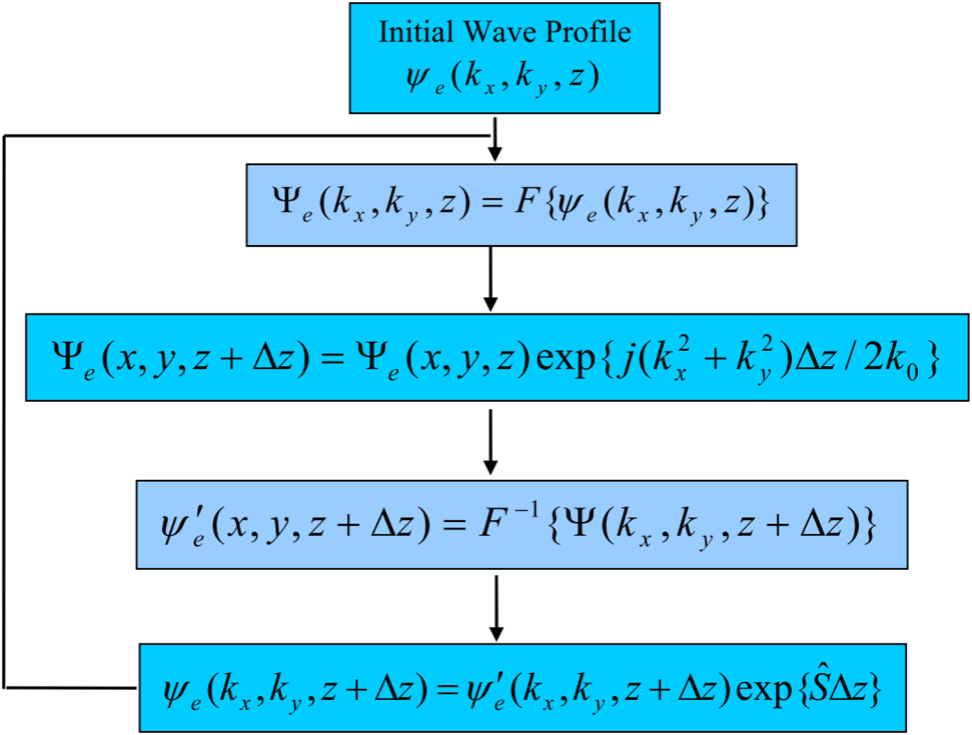
A schematic representation of the Fourier method.

Light propagation within the hydrogel structure was simulated using the Fourier method. A schematic representation of the Fourier algorithm is given in the Fig. 1, where the field alternately propagates through diffraction (in the frequency domain) and the GRIN medium (in the spatial domain) using the split-step Fourier method.

The algorithm begins with an initial profile *ψ*_e_(*k*_*x*_, *k*_*y*_, *z*) in the spatial frequency domain. A Fourier transform *F* is applied to obtain *Ψ*_e_(*k*_*x*_, *k*_*y*_, *z*). The next step is performed by multiplying the spectrum by a quadratic phase factor exp(*j*(*k*_*x*_^2^ + *k*_*y*_^2^)Δ*z*/(2*k*_0_)) to obtain *Ψ*_e_(*x*, *y*, *z* + Δ*z*). Then, an inverse Fourier transform *F*^−1^ is applied to get 
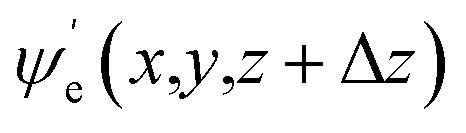
 in the spatial domain. Finally, the inhomogeneous operator exp(*S*Δ*z*) (which *S* accounts for refractive index variations) multiplies the field to produce the final propagated field *ψ*_e_(*k*_*x*_, *k*_*y*_, *z* + Δ*z*), which is again represented in the frequency domain for the next iteration. These steps are repeated to move the beam forward step by step. In each step, the beam first propagates through free space (diffraction) using Fourier transforms, and then the effect of the material (refractive index) is added in the spatial domain. Also, summary of simulation parameters used in the calculations are listed in the Table 1.

**Table 1:** Numerical data used in the simulations

Parameter	Variable	Value	Notes
Transverse window	*L*	10 mm	Size of computational domain in *x*–*y* plane
Grid size	*N* × *N*	250 × 250	Number of transverse sampling points
Spatial resolution	Δ*x* = *L*/*N*	0.04 mm	Distance between adjacent grid points
Propagation step	d*z*	0.01 mm	Step size along propagation direction (*z*)
Total propagation distance	*Z*	100 mm	Total simulated length along *z*
Number of steps	*Z*/d*z*	10 000	Total iterations
On-axis refractive index	*n*_0_	1.5	Refractive index at the center of the waveguide (*r* = 0)
Quadratic gradient coefficient	*n*_2_	0.01	Defines the parabolic refractive index profile: *n*(*r*) = *n*_0_ − 1/2*n*_2_*r*^2^
Gaussian beam waist	*ω*_0_	0.5	Initial beam waist radius at *z* = 0

**Fig. 2:**
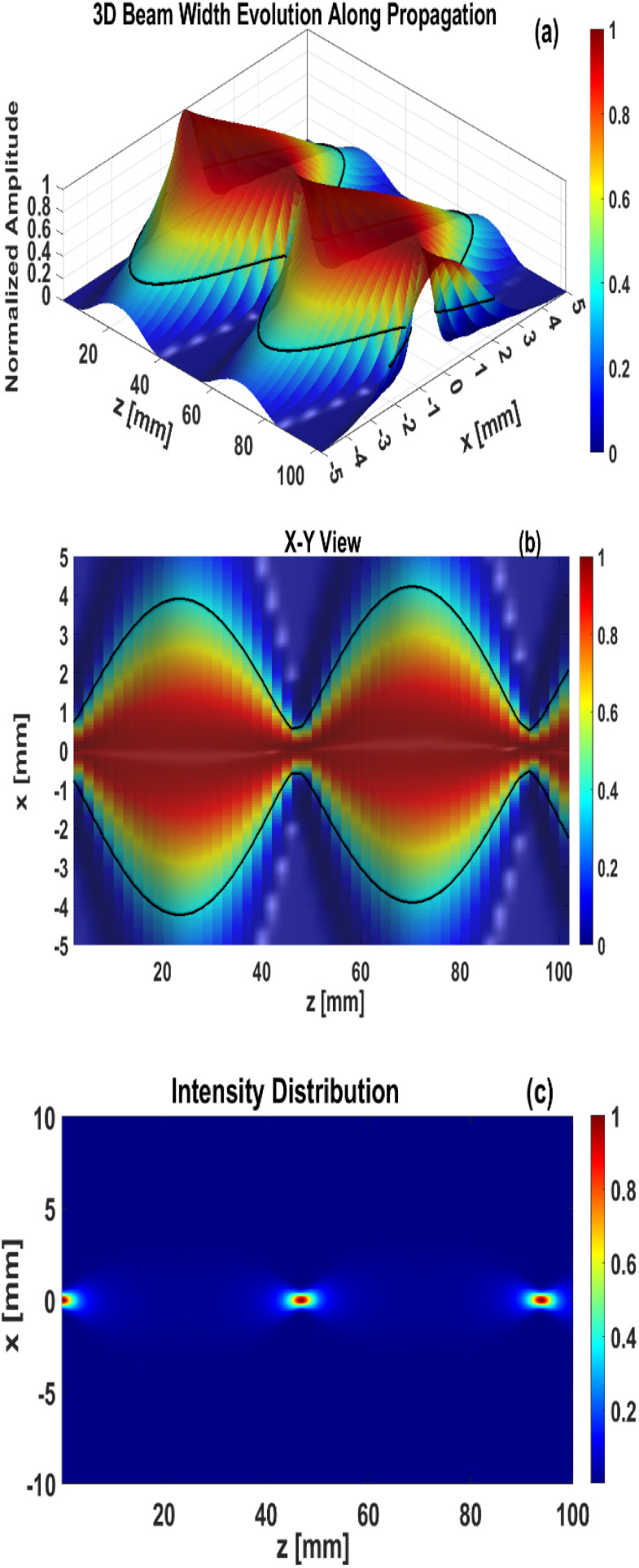
(a) 3D evolution of Gaussian beam launched into parabolic index medium beam. (b) *x*–*y* view of plot (a) and (c) intensity distribution. The vertical axis is the transverse position, *x*, and the horizontal axis is the propagation distance, *z*.

Fig. 2 illustrates the periodic focusing and defocusing of a Gaussian beam as it propagates through a soft GRIN hydrogel. Panel (a) shows the three-dimensional evolution of the normalized amplitude, where the beam repeatedly narrows and widens along the propagation distance *z*. Panel (b) presents the corresponding *x*–*y* view, which confirms that the beam remains radially symmetric at all propagation distances. Panel (c) shows the corresponding intensity distribution. From the simulations, the beam starts with an initial waist of *ω*_0_ = 0.5 mm at *z* = 0, narrows to a minimum waist of approximately 0.5 mm again at the first focal plane, and then expands. The longitudinal modulation period is given by *z*_m_ = π*h*, where *h* = *n*_0_/√*n*_2_. With *n*_0_ = 1.5 and *n*_2_ = 0.01 mm^−2^, the calculated period is *z*_m_ ≈ 47 mm, which matches the observed distance between successive focal planes in the figure. The peak normalized amplitude reaches nearly 1.0 at each focal plane and drops to about 0.4 at the defocus planes, directly confirming the periodic energy concentration predicted by eqn (6).

Here, no energy loss is considered. However, we made this simplification for three reasons. First, before adding real-world complications, we first need to show that the idea works in the ideal case. This is the standard first step in any physics or engineering study. Second, hydrogels naturally have very low scattering because their pore size is much smaller than light wavelengths.^22^ Third, scattering and absorption mainly reduce light intensity, but they do not destroy the periodic phase resetting behavior, which is the main study of our work.

**Fig. 3:**
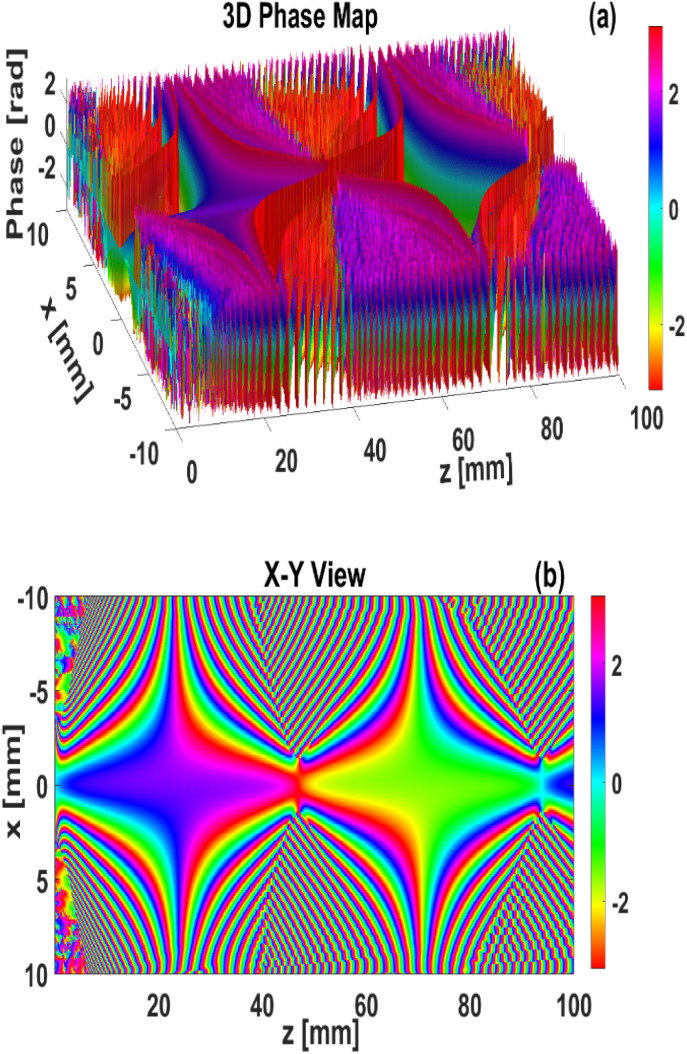
(a) Axial phase evolution *φ*(*z*) of Gaussian beam propagation in the engineered parabolic GRIN hydrogel. (b) *x*–*y* view of plot (a). The vertical axis is the transverse position, *x*, and the horizontal axis is the propagation distance, *z*.

Fig. 3 shows a fundamentally different longitudinal pattern, where periodic focusing and defocusing cycles replace monotonic spreading due to the harmonic optical potential created by the quadratic refractive index profile. In the 3D phase map (Fig. 3a), the color changes from blue (between 0 and 4 mm) to green (between 4 and 9 mm), and this pattern repeats. The repetition of this color cycle directly visualizes the periodic phase dynamics. At the focal point (about *z* = 50 mm), the phase jumps from positive to negative values. Before 50 mm, the phase is positive; after 50 mm, it becomes negative (see Fig. 3b). This jump repeats at each focal point along the propagation path. This periodic behavior is an inherent property of wave propagation in parabolic-index media.

**Fig. 4:**
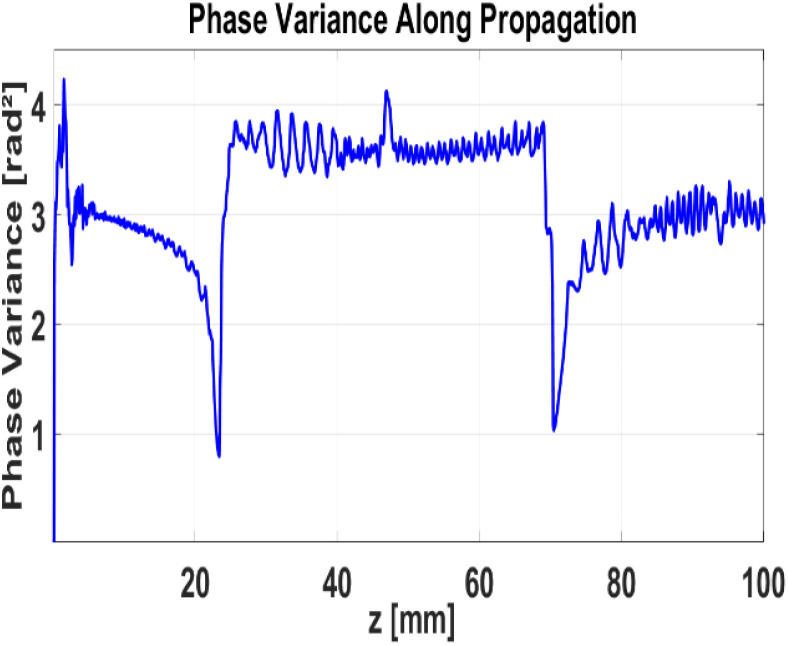
Phase variance as a function of propagation distance *z* inside the GRIN hydrogel.

The graph presented in this Fig. 4 illustrates the evolution of phase variance along the propagation distance *z* inside the GRIN medium. Our numerical results prove that the graph repeats the same cycle over and over. Each cycle starts with a decrease in phase variance. Then, variance rises sharply, stays flat for some distance, and finally decreases again. This pattern continues as the beam propagates through the hydrogel. During the flat region, the beam is propagating in a steady state between two consecutive focal points where the wavefront curvature and intensity profile change very slowly or linearly, so the phase variance does not change significantly. The phase variance pattern helps identify optimal implantation depths: avoid focal points where variance is high, and place neural targets in flat regions where variance is low and stable, ensuring sharp, predictable light focusing.

**Fig. 5:**
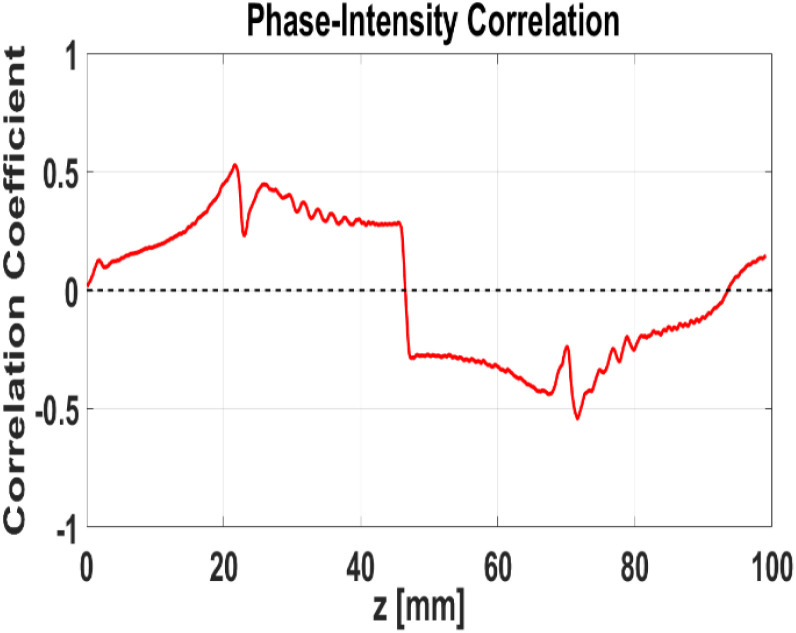
Correlation coefficient between the optical phase and the intensity of the Gaussian beam *versus* propagation distance *z*.

Fig. 5 displays the correlation coefficient between the optical phase and the intensity of the Gaussian beam *versus* propagation distance *z* inside the GRIN hydrogel. As one can see from Fig. 5, within the first 20 mm, the coefficient rises rapidly, indicating that the GRIN medium quickly establishes a deterministic phase–intensity relationship. Between 20 mm and 45 mm, the coefficient remains stable at a moderate level. At approximately *z* = 50 mm, the correlation drops sharply, which corresponds to the first focal plane. Beyond 50 mm, the coefficient partially recovers and stabilizes again, confirming that the beam regains coherence after passing through the focus. Physically, a high correlation means that at every point of the beam, the relationship between the phase front curvature and the intensity value is predictable and constant. For optogenetics, a stable phase-intensity correlation keeps the light spot sharp and focused, reducing off-target activation. Thus, the GRIN hydrogel locks phase to intensity for passive beam stabilization.

**Fig. 6:**
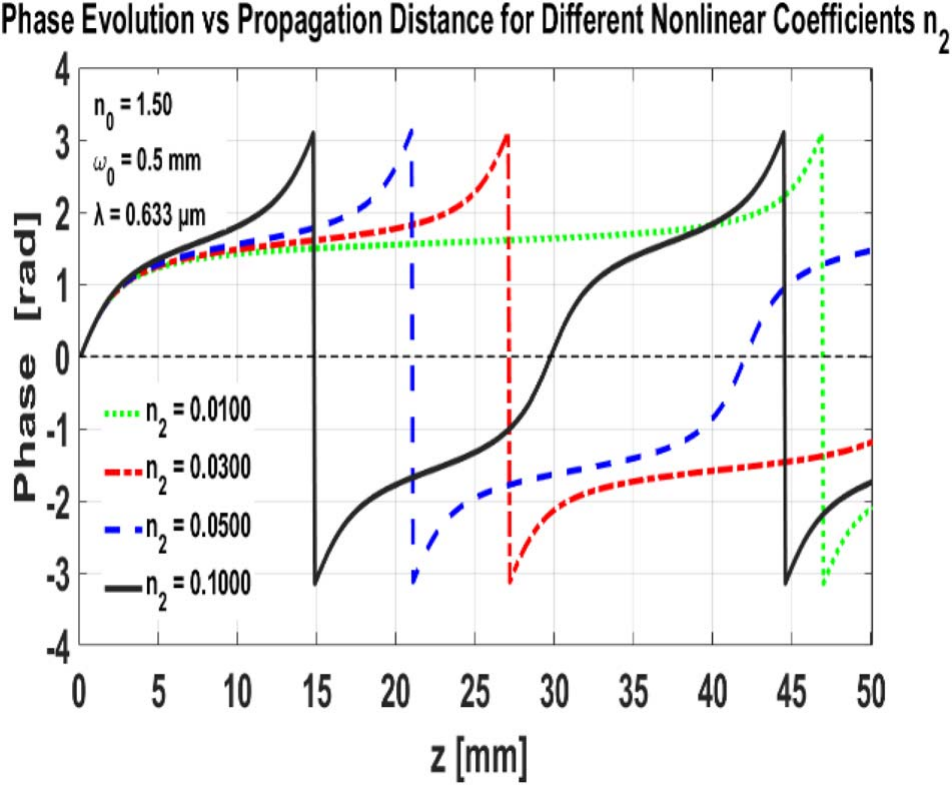
Phase evolution for four different gradient coefficients: *n*_2_ = 0.01, 0.03, 0.05, and 0.1 mm^−2^.

Fig. 6 presents the axial phase accumulation *φ*(*z*) as a function of propagation distance *z* for four different gradient coefficients: *n*_2_ = 0.01, 0.03, 0.05 and 0.10 mm^−2^. For all coefficients, the phase increases monotonically with *z*, but the slope d*φ*/d*z* becomes significantly steeper as *n*_2_ grows. The phase *φ*(*z*) includes a term proportional to (*m* + *n* + 1)*z*/*h*, where *h* = *n*_0_/√*n*_2_. Thus, for higher *n*_2_, *h* becomes smaller, and the modal contribution to the phase grows more rapidly with *z*. The phase periodically drops from +3 rad to −3 rad and then rises back from −3 rad to +3 rad, repeating this cycle continuously. A larger *n*_2_ creates a deeper harmonic potential, which may be useful for shallow targets or phase-sensitive stimulation protocols. In contrast, a lower *n*_2_ provides a slower, more gradual phase accumulation, making it suitable for deeper targets where longer, repeated focusing cycles are required. This phase resetting behavior ensures that the beam refocuses at regular intervals, making the GRIN hydrogel a predictable passive waveguide for optogenetic stimulation.

## 4 Conclusion

In this study, we numerically demonstrated that a soft parabolic-index (GRIN) hydrogel can passively stabilize the phase of a low-power Gaussian beam for optogenetic neural stimulation without any external power. Using the beam propagation method with *n*_0_ = 1.5, *n*_2_ = 0.01 mm^−2^, *λ*_0_ = 0.633 µm, and *ω*_0_ = 0.5 mm, we found that the beam propagates with a periodic focusing/defocusing cycle of period *z*_m_ = 47 mm. The normalized amplitude reaches 1.0 at each focal plane and drops to approximately 0.4 at defocus planes. The optical phase resets periodically from +3 rad to −3 rad at each focal point, confirming passive phase stabilization. The phase-intensity correlation stabilizes within the first 2 mm of propagation, ensuring predictable focusing. We also showed that increasing the gradient coefficient to *n*_2_ = 0.1 mm^−2^ accelerates phase accumulation, making it suitable for shallow targets, while lower *n*_2_ = 0.01 mm^−2^ provides slower phase cycling for deeper brain regions. These results confirm that GRIN hydrogels act as passive, biocompatible waveguides that resolve the three major challenges in optogenetics: light divergence, tissue damage from rigid fibers, and reliance on external power. This approach thus paves the way for safe, implantable, and long-lasting optogenetic probes for treating neurological disorders such as Parkinson's disease and epilepsy.

## Author contributions

H. Rahimi: writing – review & editing, writing – original draft, visualization, validation, software, project administration, methodology, investigation, formal analysis, conceptualization.

## Conflicts of interest

There are no conflicts to declare.

## Data Availability

The data that support the findings of this study are available from the corresponding author upon reasonable request.
